# Enhancement of Solvent Resistance of Polyimide Electrospun Mat via the UV-Assisted Electrospinning and Photosensitive Varnish

**DOI:** 10.3390/polym11122055

**Published:** 2019-12-11

**Authors:** Lin Qi, Chen-Yu Guo, Meng-Ge Huang Fu, Yan Zhang, Lu-meng Yin, Lin Wu, Jin-gang Liu, Xiu-min Zhang

**Affiliations:** 1 Beijing Key Laboratory of Materials Utilization of Nonmetallic Minerals and Solid Wastes, National Laboratory of Mineral Materials, School of Materials Science and Technology, China University of Geosciences, Beijing 100083, China; 2103180030@cugb.edu.cn (L.Q.); 2103170019@cugb.edu.cn (C.-Y.G.); 2103180029@cugb.edu.cn (M.-G.H.F.); 2103170021@cugb.edu.cn (Y.Z.); 2003180030@cugb.edu.cn (L.-m.Y.); 2003190023@cugb.edu.cn (L.W.); 2 School of Electrical Engineering, Beijing Jiaotong University, Beijing 100044, China

**Keywords:** electrospinning, photosensitive polyimide, ultrafine fibrous mat, solvent resistance

## Abstract

A new methodology for enhancing the solvent resistance of electrospun polyimide (PI) ultrafine fibrous mat (UFM) was investigated in the current work. For this purpose, a negative intrinsically photosensitive polyimide (PSPI) resin was prepared by the one-step high- temperature polycondensation procedure from 3,3’,4,4’-benzophenonetetracarboxylic dianhydride (BTDA) and α,α-bis(4-amino-3,5-dimethylphenyl)phenylmethane (PTMDA). The PI varnish, by dissolving the derived PI (BTDA-PTMDA) resin in *N,N*-dimethylacetamide (DMAc) at a solid of 20 wt %, was used as the starting material for the standard electrospinning (ES) and ultraviolet-assisted ES (UVAES) fabrications, respectively. The 365 nm wavelength of the high-pressure mercury lamp ultraviolet (UV) irradiation induced the photocrosslinking reaction in the PSPI mat. Solubility tests indicated that the PI UFM fabricated by standard ES procedure showed poor DMAc resistance, while the one by UVAES (PI-UV) exhibited excellent resistance to DMAc.

## 1. Introduction

Electrospinning (ES) fabrication has been thought to be one of the most effective procedures for producing polymeric ultrafine fibrous mats (UFMs) [1]. In recent years, the ever-increasing demands of high performance polymeric UFMs in high-tech fields has greatly accelerated the research and development of new ES products and advanced ES techniques [2]. For example, various electrospun polyimide (PI) UFMs with excellent combined properties, including high thermal stability, good environmental stability, high specific surface areas, and other desired properties have been developed [3,4,5,6,7,8,9,10]. PI UFMs have exhibited great potential as high performance filtration components for air purification [11,12], hot oil filtration [13], low fouling microfiltration for biorefinery fields [14], and other advanced applications.

As for the fabrication procedures for PI UFMs, standard ES techniques have been successfully achieved either with the conventional PI precursors, poly(amic acid)s (PAAs) or organo-soluble preimidized PI solution as the starting materials. For the former procedure utilizing PAAs as the spinning agents, an additional high temperature imidization procedure with the final temperature up to 350 °C has to be carried out, in which the PAA precursors were thermally dehydrated to afford the insoluble PI UFMs. The PI UFMs fabricated by such procedure usually exhibit excellent solvent resistance and high mechanical strength. However, they usually suffer from yellowing, caused by the high-temperature imidization environments [15,16]. In addition, the fusion between the individual fiber in the PI UFMs and the pinholes formed in the course of water molecules liberation during the imidization reaction often deteriorate the physical and chemical properties of the PAA-type PI UFMs [17]. Alternatively, fabrication of electrospun PI UFMs with preimidized PI solution has been paid ever-increasing attention, due to the advantages of low-temperature curability, pale-color appearance, and perfect fiber surface without pinholes or potholes caused by the liberation of water in the imidization course for PAA system [18]. However, the poor resistance of such PI UFMs to organic solvents, especially polar aprotic solvents is a major challenge in their applications, such as purification and separation of high-temperature polar organic fluids and so on [13]. In order to enhance the solvent resistance of soluble PI UFMs while maintaining the intrinsic merits for such materials, new soluble PIs and ES procedures are highly desired.

It has been well established that chemical crosslinking is one of the most useful and effective procedures for enhancing the solvent resistances of polymers [19]. Actually, crosslinking PIs have been widely used in the mat applications [20]. Among the various crosslinking methodologies, photocrosslinking might be more suitable for the solvent resistance of PI UFMs. This is mainly due to the high specific surface area and thin electrospun fiber, which might provide sufficient contact of the fiber with the UV irradiation. Highly-efficient photocrosslinking reaction can be expected to be attained.

In the current work, with the aim of enhancing the solvent resistance of soluble PI UFMs, the feasibility of UV-assisted electrospinning (UVAES) with photo-curable PI spinning solution as the starting materials was evaluated. For this purpose, an auto-photosensitive PI (PSPI) or intrinsically PSPI based on 3,3’,4,4’-benzophenonetetracarboxylic dianhydride (BTDA) and *ortho*-alkyl- substituted diamine was prepared. This series of PSPIs was first reported by OCG Microelectronic Materials, Inc., Switzerland in 1985 [21,22]. Such preimidized soluble PSPIs are photosensitive to the wavelength of 365 nm ultraviolet light without adding any additional photosensitizers (intrinsically photosensitive); thus exhibiting relatively small volume shrinkage and thickness loss of the PIs in the photolithography process. Conversely, the intrinsically PSPIs exhibit a negative working mechanism in the photolithography procedure; that is the UV-irradiated area undergoes photo-crosslinking reactions and becomes insoluble in the developing agents, such as *N,N*-dimethylformamide [23]. The influence of the UVAES procedure on the solubility, thermal, and optical properties of the PSPI UFMs was investigated in detail.

## 2. Materials and Methods

### 2.1. Materials

3,3’,4,4’-Benzophenonetetracarboxylic dianhydride (BTDA) was purchased from Evonik Degussa Corp. (Los Angeles, CA, USA) and dried in vacuo for 24 h at 180 °C prior to use. The aromatic diamine, α,α-bis-(4-amino-3,5-dimethylphenyl)phenylmethane (PTMDA) was synthesized in our laboratory according to the literature [24]. High purity solvents including *N*-methyl-2-pyrrolidinone (NMP) and *N,N*-dimethylacetamide (DMAc) were all purchased from Tokyo Chemical Industry (Tokyo, Japan) and further purified by distillation prior to use. The other commercially available reagents were used without further purification.

The high-pressure mercury lamp (power: 100W) with the maximum emitting wavelength of 365 nm was purchase from BILON Company (Shanghai, China) and equipped with a quartz cold trap.

### 2.2. Preparation of PSPI resin

**Scheme 1:**
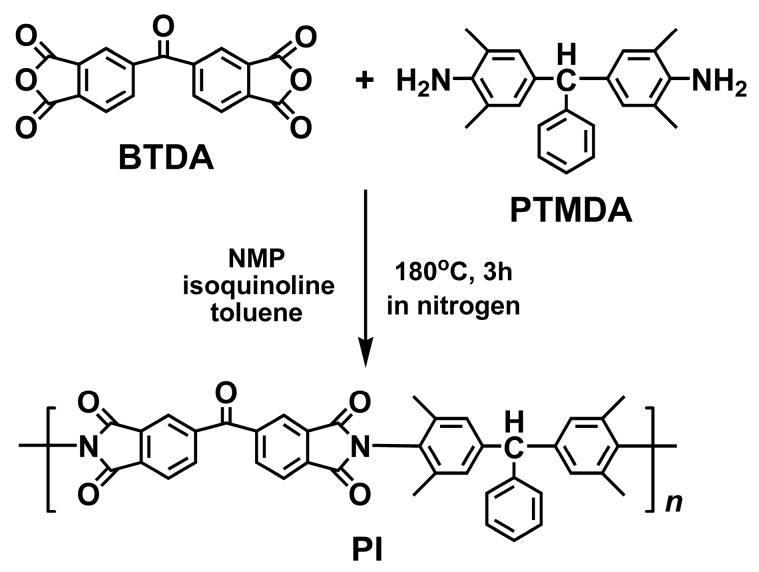
Synthesis of polyimide (PI) (BTDA-PTMDA).

As shown in Scheme 1, the PI resin was prepared by the one-step high-temperature polycondensation procedure of BTDA and PTMDA using NMP as the solvent and isoquinoline as the catalyst. The procedure for the synthesis of PI resin is as follows. Into a 500 mL three-necked, round-bottomed flask equipped with a mechanical stirrer, a Dean–Stark trap and a nitrogen inlet, PTMDA (33.0470 g, 100 mmol) was dissolved in NMP (197 g) to give a clear solution. Then, BTDA (32.2230 g, 100 mmol) was added in one batch and an additional volume of NMP (100 g) was added to wash the residual dianhydride, and at the same time to adjust the solid content of the reaction system to be 18 wt %. Isoquinoline (0.5 g) was added as an imidization catalyst. After stirring in nitrogen for 1 h, toluene (100 mL) was then added as an azeotropic agent. The reaction mixture was heated to 180 °C and maintained for 3 h. During the reaction, the toluene–water azeotrope was distilled out of the system and collected in the Dean–Stark trap. After cooling to room temperature, the viscous solution was carefully poured into an excess of ethanol to yield a silky brown resin. The obtained PI resin was collected and dried at 80 °C in vacuum for 24 h. The yield was 59.2 g (96%).

Pale-brown PI resin was obtained quantitatively from the PI solution by precipitation in excess of ethanol. Actually, the first generation of such auto-sensitive PSPI resins are based on BTDA and 2,3,5,6-tetramethyl-1,4-phenylenediamine (TMPDA) or 3,3’,5,5’-tetramethyl-4,4’-diaminodiphenyl- methane (TMMDA) [23]. In view of the relatively limited solubility of the first generation of products in organic solvents, a new generation of highly soluble intrinsic PSPI was developed in our laboratory [24].

### 2.3. Fabrication of PSPI Electrospun Fibrous Mats

#### 2.3.1. Standard Electrospinning 

The well-dried PI resin (2.0 g) was dissolved in dry DMAc (8.0 g) at room temperature with a solid content of 20 wt %. The absolute viscosity of the obtained PI solution was 8154 mPa·s. The pristine PI solution was filtered through a 0.45 µm polytetrafluoroethylene (PTFE) filter to remove any impurities that might block the spinneret. Part of the PI solution was filled into a 5 mL syringe and pumped through the fine hole of a spinneret by a syringe pump at a speed of 0.1 mL/h. The inner diameter of the spinneret is 0.5 mm. A voltage of 15 kV was applied between the syringe and the rolling drum collector. The distance between the spinneret and the grounded collector was 15 cm. Random aligned PI fibers were deposited on the aluminum foil as the support medium of the fibers, located on the rotating drum collector (diameter: 10 cm; length: 30 cm; speed: 200 rpm). The humidity in the electrospinning apparatus was controlled to be 50 ± 2% relative humidity. The obtained PI fibrous mat was dried at 200 °C for 1 h to remove the residual solvent.

#### 2.3.2. UV-assisted Electrospinning (UVAES)

**Figure 1:**
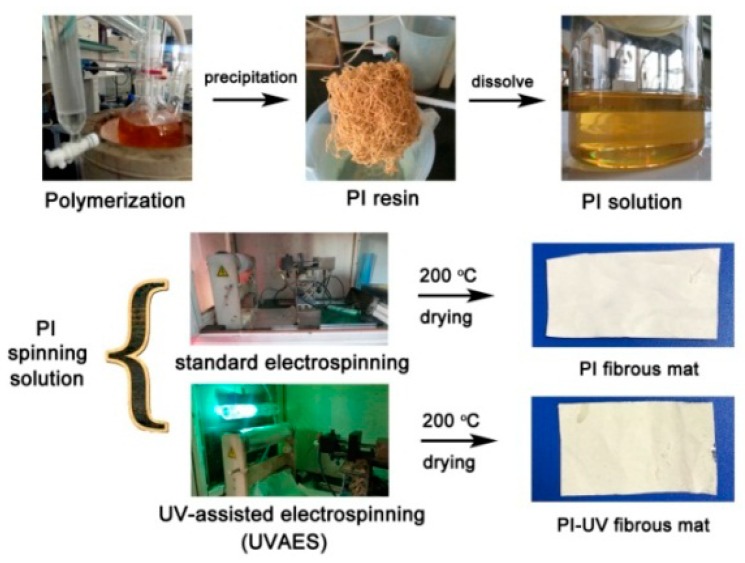
Preparation process for PI and PI-UV fibrous mats.

The electrospinning process was similar to that of the standard one except, that a high-pressure mercury lamp (*λ*_max_: 365 nm) equipped with a cold bath with the set temperature of 5–6 °C was placed on the top of the rolling drum collector (Figure 1). The distance between the UV lamp and the collector was 5 cm. The UV exposure was continuous in the whole electrospinning fabrication.

### 2.4. Characterization Methods

Absolute viscosity was measured using a Brookfield DV-II+ Pro viscometer (Brookfield Ametek, Middleboro, MA, USA) at 25 °C. Inherent viscosity was measured using an Ubbelohde viscometer (Mitong Electromechanical Tech. Co. Ltd., Shanghai, China) with a 0.5 g/dL NMP solution at 25 °C. The average molecular weight (*Mn*) and weight average molecular weight (*Mw*) of the PI resins were measured using a gel permeation chromatography (GPC) system (Shimadzu, Kyoto, Japan) with a LC-20AD dual-plunger parallel-flow pumps (D1-LC), a SIL-20A is a total-volume injection-type autosampler, a CTO-20A column oven, and a RID-20A detector. HPLC grade NMP was used as the mobile phase at a flow rate of 1.0 mL/min. Attenuated total reflectance Fourier transform infrared (ATR-FTIR) spectrum was obtained on a Tensor-27 FT-IR spectrometer (Bruker, Ettlingen, Germany). Nuclear magnetic resonances (^1^H-NMR) were performed on an AV 400 spectrometer (Bruker, Ettlingen, Germany) operating at 400 MHz in DMSO-d6. Wide-angle X-ray diffraction (XRD) was conducted on a Rigaku D/max-2500 X-ray diffractometer (Tokyo, Japan) with Cu-Kα1 radiation, operated at 40 kV and 200 mA. Field emission scanning electron microscopy (FE-SEM) was carried out using a JSM-6700F (JEOL, Tokyo, Japan) with an accelerating voltage of 15 kV for imaging. Pt/Pd was spattered on each film in advance of the measurements. Ultraviolet-visible (UV-Vis) spectra were recorded on a U-3210 spectrophotometer (Hitachi, Tokyo, Japan) at room temperature. Prior to test, PI mats were dried at 100 °C for 3 h to remove the absorbed moisture. The whiteness of the PI fibrous mat was measured using an X-rite color i7 spectrophotometer (Grand Rapids, MI, USA) in accordance with the procedure described in Chinese standard GB/T 17644-2008 (test method for whiteness and chromaticity of textile fibers) and in the standard of ISO 11475: 2017 (paper and board, determination of CIE whiteness, D65/10°, outdoor daylight). The color parameters were calculated according to a CIE Lab equation. L* is the lightness, where 100 means white and 0 implies black. A positive a* means a red color, and a negative one indicates a green color. A positive b* means a yellow color, and a negative one indicates a blue color. The whiteness values of the PI mats were calculated as Equation (1), where *WI* standards for whiteness index, L* standards for index of lightness, a* and b* stand for chromaticity coefficient.

*WI* = 100 − [(100 - L*)^2^ + a*^2^ + b*^2^]^1/2^ (1)

Thermogravimetric analysis (TGA) was performed on a TA-Q50 thermal analysis system (New Castle, DL, USA) at a heating rate of 20 °C/min in nitrogen with the maximum temperature of 760 °C in the heating scan. Differential scanning calorimetry (DSC) was carried on a TA-Q 100 thermal analysis system (New Castle, DL, USA) at a heating rate of 10 °C/min in nitrogen with the maximum temperature of 400 °C in the heating scan.

Solubility of the PI fibrous mats fabricated by standard electrospinning procedure (PI) and the UV-assisted electrospinning (UVAES) procedure (PI-UV) were measured and compared by immersing the free-standing PI mats (Length: 50 mm; Width: 30 mm; Thickness: 1 mm) into the pure DMAc contained in a glass vessel. The solubility of the fibrous mats was visually observed.

## 3. Results and Discussion

**Figure 2:**
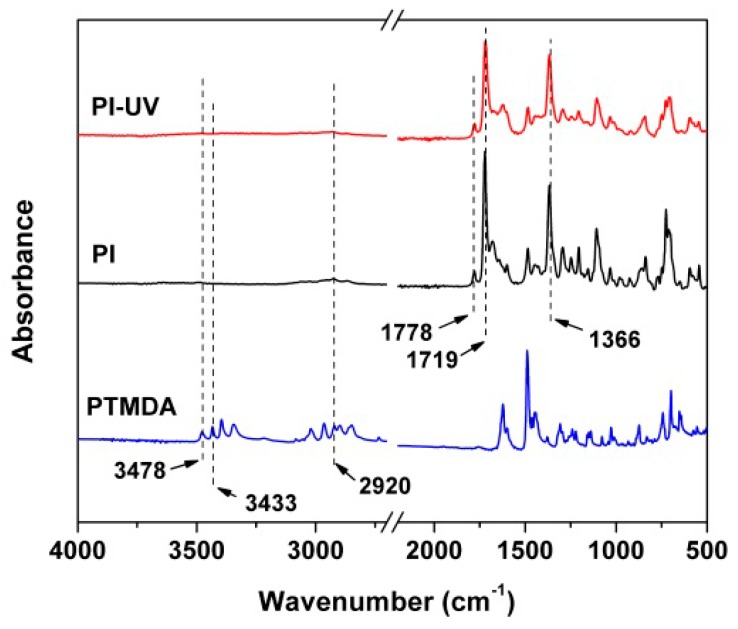
ATR-FTIR spectra of PTMDA and the derived PI mats.

**Figure 3:**
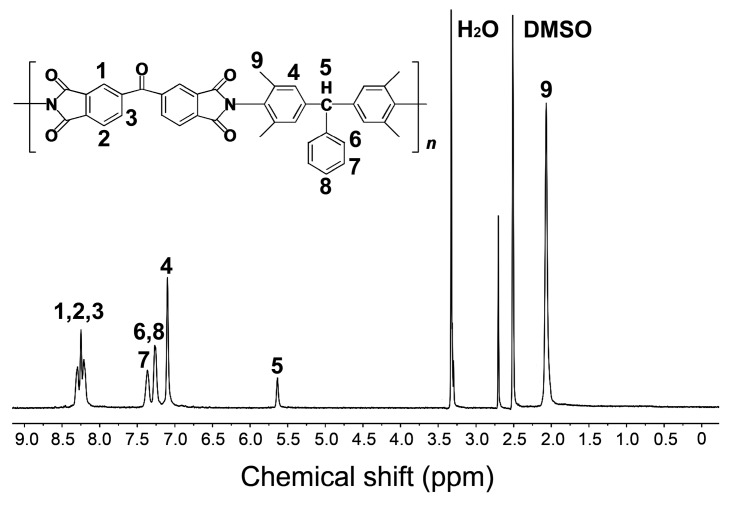
^1^H-NMR spectrum of PI (BTDA-PTMDA) resin.

The chemical structure of the PI and PI-UV UFMs were confirmed by Fourier transform infrared spectrum (FT-IR) and nuclear magnetic resonances (NMR) measurements. Figure 2 compares the attenuated total reflectance FT-IR (ATR-FTIR) spectra of the PIs together with that of the PTMDA monomer. On the one hand, the characteristic absorption of primary –NH_2_ in PTMDA at 3478 cm^−1^ and 3433 cm^−1^ all disappeared in the spectra of PI and PI-UV, while those of the imide ring, including the asymmetric stretching vibrations of carbonyl at 1778 cm^−1^, symmetric stretching vibrations of carbonyl at 1719 cm^−1^, and C–N stretching vibration at 1366 cm^−1^ were clearly observed for both of PI and PI-UV. This indicates the successful preparation of the PIs. The chemical structure of the PI could be further identified by the nuclear magnetic resonances (NMR) measurement. Figure 3 shows the ^1^H-NMR spectrum of PI (BTDA-PTMDA), in which all of the hydrogen protons could be clearly assigned.

**Figure 4:**
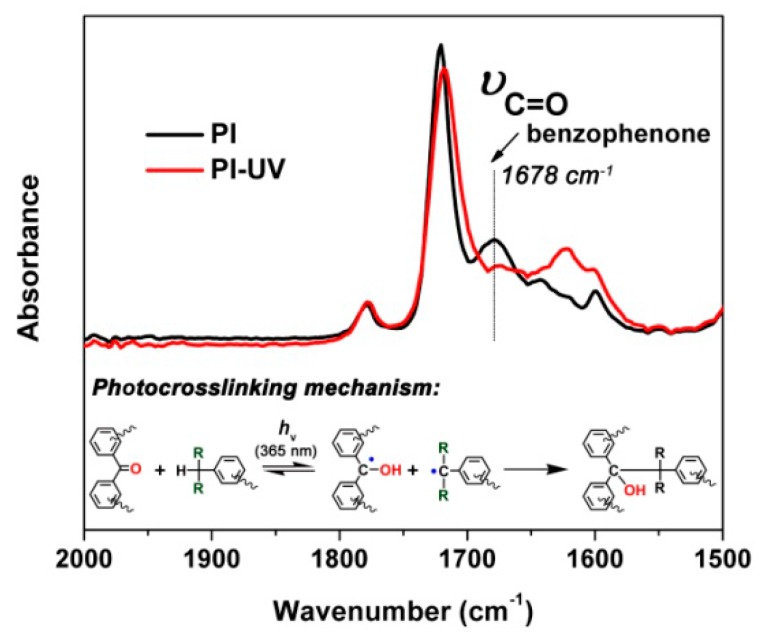
ATR-FTIR spectra of PI and PI-UV mats in the range of 2000–1500 cm^−1^. (Inserted: photocrosslinking mechanism of the PI).

The ATR-FTIR spectra of the PI and PI-UV mats also provided information on the photocrosslinking mechanism of the PI (BTDA-PTMDA). It has been well established that the presence of both of the benzophenone units in the dianhydride moiety and the hydrogen donors in the diamine moiety are contributable to the photocrosslinking of such auto-sensitive PIs [25,26]. Upon irradiation to the 365 nm UV light, the triplet benzophenone units undergo hydrogen abstraction from the adjacent *ortho*-substituted alkyl groups in the diamine moiety. The formed radicals react with each other to yield the crosslinking structure. This mechanism can be proven by comparing the characteristic absorption of carbonyl in benzophenone unit before and after UV irradiation, as illustrated in Figure 4. The wavenumber of 1678 cm^−1^ represents the characteristic absorption of >C=O in benzophenone, whose absorption strength obviously decreased in the spectrum of PI-UV (red line), indicating that the UV irradiation obviously decreased the contents of benzophenone in the polymer. According to the measurements, the photocrosslinking mechanism for the auto-sensitive PI (BTDA-PTMDA) was inserted in the figure. Interestingly, the efficiency of this photochemistry reaction for the auto-sensitive PI is so high that it is beyond our imagination. Generally, for thin film materials, the photocrosslinking reaction usually occurs in a very limited depth range on the surface of the film. With the progress of the crosslinking reaction, the UV light penetration often decreases sharply and the crosslinking reaction decreases dramatically. In this study, the specific surface area of the electrospun fiber is very large and the fiber diameter is quite small, so the photocrosslinking reaction efficiency is very high, and the reaction occurs efficiently in a quite short time from the spinneret head to the collector. The absorption intensity of the benzophenone carbonyl in the molecular structure of PI decreased dramatically. The high efficient combination of the UV and ES techniques provides a new way to develop high performance electrospun PI materials.

**Figure 5:**
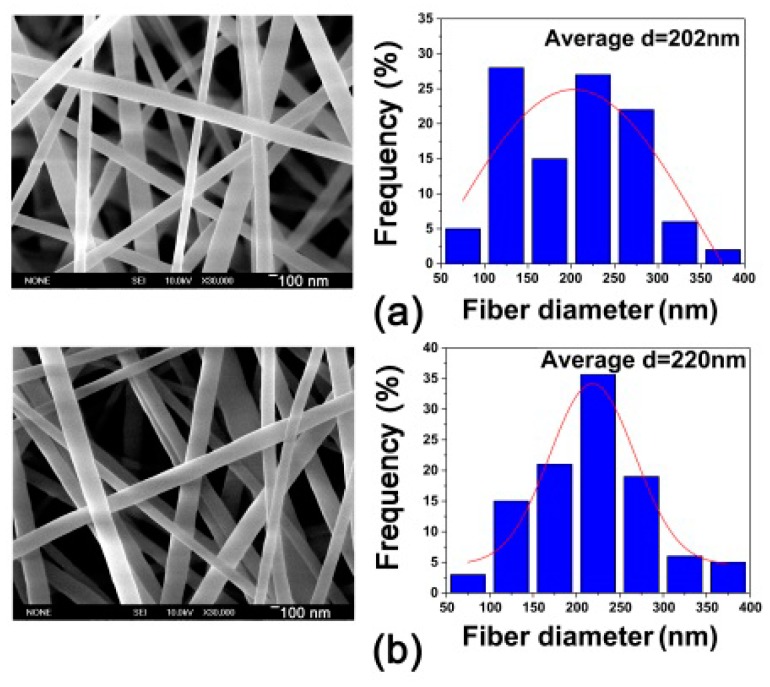
FE-SEM images and fiber diameters of PI fibrous mats (**a**) PI; (**b**) PI-UV.

The micro-morphography of the PI UFMs was observed by field emission scanning electron microscopy (FE-SEM) and the images are shown in Figure 5. It can be clearly observed from the micro-morphology of the PI UFMs that the ultrafine non-woven fibrous mats with spatial reticulated structures were obtained either by conventional ES process (Figure 5a) or by the UVAES process (Figure 5b). The UV crosslinking has no significant effect on the shape of the fiber, but the average diameters of the fiber increased from 202 nm of the PI UFM to 220 nm of PI-UV UFM.

**Figure 6:**
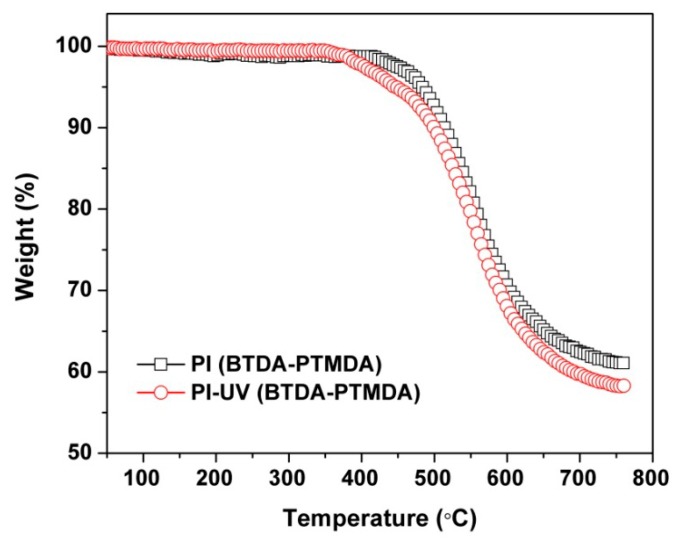
TGA curves of PI and PI-UV fibrous mats.

**Figure 7:**
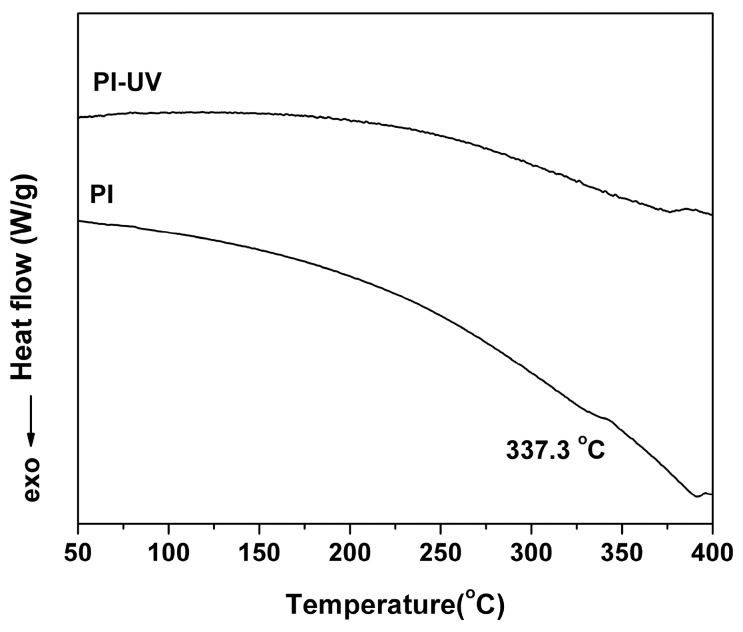
DSC curves of PI and PI-UV fibrous mats.

**Figure 8:**
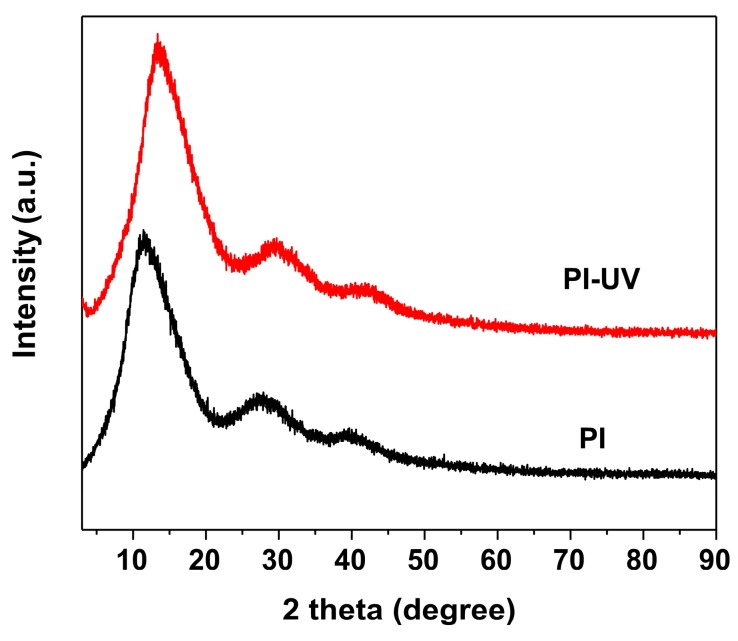
XRD plots of PI and PI-UV fibrous mats.

The thermal properties of the PI and PI-UV UFMs were investigated by thermogravimetric analysis (TGA) and the results are shown in Figure 6 and Table 1. UV crosslinking slightly decreased the thermal stability of the PI UFMs. The initial thermal decomposition temperature (*T*_5%_) of PI-UV UFM is 447.2 °C, which is 36.5 °C lower than that of the PI UFM. This deterioration in thermal stability of the PI might be due to the formation of benzhydrol unit (C–OH, shown in Figure 4) in the crosslinked PI, the thermal stability of which was inferior to that of the benzophenone moiety [27]. The glass transition behaviors of the PI UFMs were evaluated by differential scanning calorimetry (DSC) measurement. Figure 7 shows the DSC plots of the PI and PI-UV UFMs and Table 1 gives the glass transition temperature (*T*_g_) data of the PIs. For the PI UFM, clear *T*_g_ was recorded at 337.3 °C; however, the glass transition disappeared in the plot of the PI-UV. This result also confirmed the occurrence of the crosslinking reaction in the PI-UV system. The microscopic reticular structure formed by the photocrosslinking inhibited the movement of the molecular chains and kept them stable at elevated temperatures. Figure 8 compares the XRD plots of the PI UFMs before and after UV irradiation. It can be clearly observed that photocrosslinking did not change the degree of crystallinity apparently and both of the PI UFMs exhibited typical amorphous nature.

**Figure 9:**
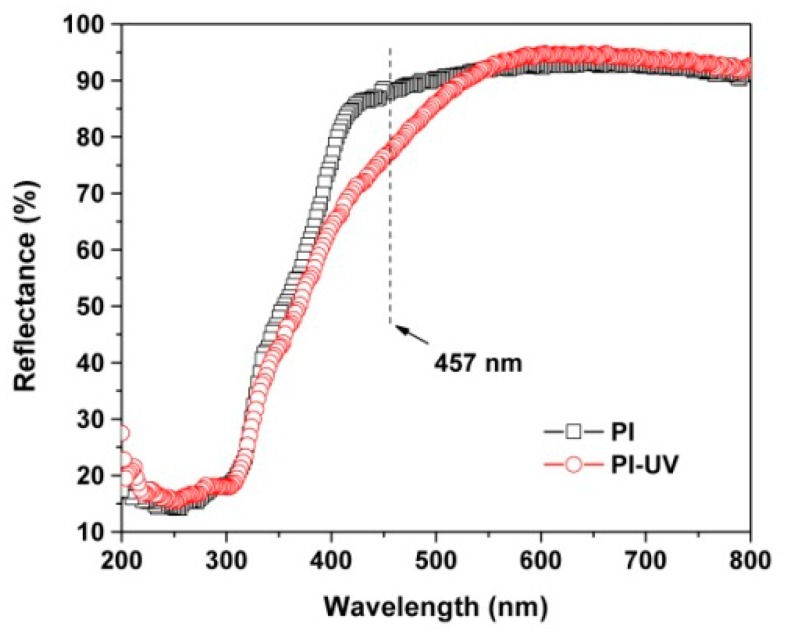
UV-Vis reflectance plots of the PI and PI UFMs.

The optical properties of the PI and PI-UV UFMs were evaluated by the ultraviolet-visible reflectance and yellow index measurements and the results are shown in Table 1. The UV reflectance data of the PIs at the wavelength of 457 nm were 88.1% for PI UFM and 77.4% for PI-UV UFM, respectively, indicating that the UV crosslinking slightly decreased the optical reflectance of the mats (Figure 9). This might be due to the increase of average fiber diameter of the PI-UV UFM caused by the photocrosslinking, which decreased the specific surface of the mat and decreased the optical reflectance at the same time. Conversely, the color change during the UV irradiation might also decrease the optical reflectance of the PI-UV UFM.

**Figure 10:**
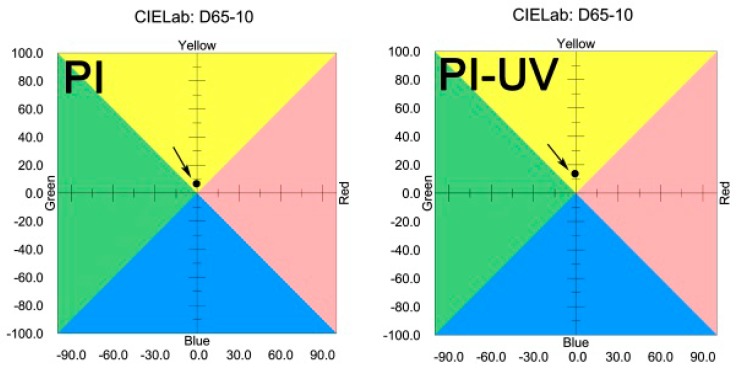
Color parameters of the PI and PI UFMs.

The color change of the PIs was quantitatively evaluated by the International Commission on Illumination (CIE) Lab measurements, in which the color parameters, including the lightness (*L*), a^*^ (red and green), b^*^ (yellow and blue), and the whiteness indices (*WI*) of the PI UFMs were detected or calculated. It can be seen from the CIE Lab image (Figure 10) and the optical data tabulated in Table 1 that UV irradiation caused yellowing of the PI mat with the b^*^ value increased from 6.7 (PI UFM) to 13.7 (PI-UV UFM). Meanwhile, the *WI* values of the PI mats slightly decreased from 90.0% of the PI UFM to 84.3% of the PI-UV mat. Basically, the optical properties of the PI UFMs were deteriorated to some extent by the UV irradiation, which is mainly attributed to the absorption of UV light and oxidation of phenyl rings in the aromatic PIs [28].

**Figure 11:**
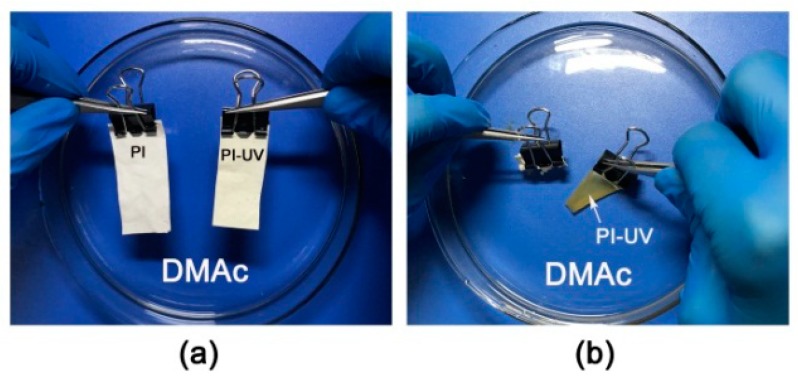
Solubility test of PI and PI-UV UFMs in *N,N*-dimethylacetamide (DMAc). (**a**) Pristine samples; (**b**) After test.

Lastly, the solvent resistance of the PI and PI-UV UFMs was investigated and compared. Figure 11 illustrates the solubility of the free-standing PI and PI-UV mats in DMAc at room temperature. It can be clearly observed that the PI mat fabricated by standard ES procedure was easily soluble in the solvent. Contrarily, the PI-UV mat maintained its original shape in the solvent although a volume shrinkage phenomenon was observed. Undoubtedly, the enhancement of solvent resistance of PI (BTDA-PTMDA) UFM is attributed to the photocrosslinking reaction in the UVAES procedure. The intra- and intermolecular crosslinked structure is beneficial for prohibiting the solvent penetration into the PIs, resulting in the improved solvent resistance of the mat.

## 4. Conclusions

In summary, the standard ES procedure was successfully combined with the UV curing technique in the current work. Taking advantage of the characteristics of large specific surface area and small diameter of electrospun fibers, it is possible to ensure contact with the UV light sufficiently in very limited time, so that the photocrosslinking reaction can be carried out efficiently. By this methodology, high performance PI UFM was fabricated from an auto-sensitive negative PSPI spinning solution. The developed PI-UV UFM exhibited enhanced solvent resistance and acceptable thermal and properties. The good comprehensive properties of the PI-UV UFMs make them good candidates as high performance filtration, separation, or other components for high-tech applications.

## Figures and Tables

**Table 1:** Properties of PI resin and PI ultrafine fibrous mats (UFMs).

PI	[*η*]_inh_^a^ (dL/g)	Molecular Weight ^b^				Thermal Properties ^c^				Optical Properties ^d^				
		*M*_n_ (g/mol)	*M*_w_ (g/mol)	PDI		*T*_g_ (°C)	*T*_5%_ (°C)	*R*_w750_ (%)		*R*_457_ (%)	*L*	a*	b*	*WI* (%)
**PI**	**1.21**	**69628**	136560	1.96		337.3	483.7	61.2		88.1	92.6	−0.3	6.7	90.0
**PI-UV**	ND ^e^	ND	ND	ND		ND	447.2	58.3		77.4	92.4	−0.6	13.7	84.3

^a^ Inherent viscosities measured with a PI resin at a concentration of 0.5 g/dL in NMP at 25 °C; ^b^ *M*_n_: number average molecular weight; *M*_w_: weight average molecular weight; PDI: polydispersity index, PDI = *M*_w_/*M*_n_; ^c^ *T*_g_: glass-transition temperatures; *T*_5%_: 5% weight loss temperatures; *R*_w750_: residual weight ratio at 750 °C in Nitrogen; ^d^ *R*_457_: reflectance of the PI UFMs at the wavelength of 457 nm; ^e^ Not detected.

## References

[1] Ahmed F.E., Lalia B.S., Hashaikeh R. (2015). A review on electrospinning for membrane fabrication: Challenges and applications. Desalination.

[2] Ray S.S., Chen S.S., Li C.W., Nguyen N.C., Nguyen H.T. (2016). A comprehensive review: Electrospinning technique for fabrication and surface modification of membranes for water treatment application. RSC Adv..

[3] Ding Y., Hou H., Zhao Y., Zhu Z., Fong H. (2016). Electrospun polyimide nanofibers and their applications. Prog. Polym. Sci..

[4] Wang Q., Song W.L., Wang L., Song Y., Shi Q., Fan L.Z. (2014). Electrospun polyimide-based fiber membranes as polymer electrolytes for lithium-ion batteries. Electrochim. Acta.

[5] Shayapat J., Chung O.H., Park J.S. (2015). Electrospun polyimide-composite separator for lithium-ion batteries. Electrochim. Acta.

[6] Miao Y.E., Zhu G.N., Hou H., Xia Y.Y., Liu T.X. (2013). Electrospun polyimide nanofiber-based nonwoven separators for lithium-ion batteries. J. Power Sour..

[7] Cao L., An P., Xu Z., Huang J. (2016). Performance evaluation of electrospun polyimide non-woven separators for high power lithium-ion batteries. J. Electroanal. Chem..

[8] Li S., Wu D., Yan X., Guan Y. (2015). Acetone-activated polyimide electrospun nanofiber membrane for thin-film microextraction and thermal desorption-gas chromatography-mass spectrometric analysis of phenols in environmental water. J. Chromatogr. A.

[9] Oktay B., Toker R.D., Kayaman-Apohan N. (2015). Superhydrophobic behavior of polyimide-siloxane mats produced by electrospinning. Polym. Bull..

[10] Gu J., Lv Z., Wu D., Guo Y., Tian L., Qiu H., Li W., Zhang Q. (2017). Dielectric thermally conductive boron nitride/polyimide composite with outstanding thermal stabilities via in-situ polymerization-electrospinning-hot press method. Compos. Part A Appl. Sci. Manuf..

[11] Zhang R., Liu C., Hsu P.C., Zhang C., Liu N., Zhang J., Cui Y. (2016). Nanofiber air filters with high-temperature stability for efficient PM2. 5 removal from the pollution sources. Nano Lett..

[12] Khalid B., Bai X., Wei H., Huang Y., Wu H., Cui Y. (2017). Direct blow-spinning of nanofibers on a window screen for highly efficient PM2. 5 removal. Nano Lett..

[13] Jiang S., Hou H., Agarwal S., Greiner A. (2016). Polyimide nanofibers by “Green” electrospinning via aqueous solution for filtration applications. ACS Sustain. Chem. Eng..

[14] Gautam A.K., Lai C., Fong H., Menkhaus T.J. (2014). Electrospun polyimide nanofiber membranes for high flux and low fouling microfiltration applications. J. Membr. Sci..

[15] Guo C.Y., Wu X., Liu J.G., Zhang Y., Zhang X.M. (2018). Preparation and Properties of High-whiteness Polyimide Ultrafine Fabrics by Electrospinning from Organo-soluble Semi-alicyclic Polyimide Resins. J. Photopolym. Sci. Technol..

[16] Guo C.Y., Liu J.G., Yin L.M., Huangfu M.G., Zhang Y., Wu X., Zhang X.M. (2018). Preparation and Characterization of Electrospun Polyimide Microfibrous Mats with High Whiteness and High Thermal Stability from Organo-soluble Polyimides Containing Rigid-rod Moieties. Fibers Polym..

[17] Goponenko A.V., Hou H., Dzenis Y.A. (2011). Avoiding fusion of electrospun 3,3′,4,4′-biphenyl- tetracarboxylic dianhydride-4,4′-oxydianiline copolymer nanofibers during conversion to polyimide. Polymer.

[18] Liu J., Huang J., Wujcik E.K., Qiu B., Rutman D., Zhang X., Guo Z. (2015). Hydrophobic electrospun polyimide nanofibers for self-cleaning materials. Macromol. Mater. Eng..

[19] Nielsen L.E. (1969). Cross-linking–effect on physical properties of polymers. J. Macromol. Sci. Part C.

[20] Vanherck K., Koeckelberghs G., Vankelecom I.F. (2013). Crosslinking polyimides for membrane applications: A review. Prog. Polym. Sci..

[21] Rohde O., Smolka P., Falcigno P.A., Pfeifer J. (1992). Novel auto-photosensitive polyimides with tailored properties. Polym. Eng. Sci..

[22] Scaiano J.C., Netto--Ferreira J.C., Becknell A.F., Small R.D. (1989). The mechanism of photocure of inherently photosensitive polyimides containing a benzophenone group. Polym. Eng. Sci..

[23] Rich D.C., Sichel E.K., Cebe P. (1996). Curing study of a preimidized photosensitive polyimide. Polym. Eng. Sci..

[24] Qian Z.G., Pang Z.Z., Li Z.X., He M.H., Liu J.G., Fan L., Yang S.Y. (2002). Photoimageable polyimides derived from α, α-(4-amino-3, 5-dimethylphenyl) phenylmethane and aromatic dianhydride. J. Polym. Sci. Part A Polym. Chem..

[25] Lin A.A., Sastri V.R., Tesoro G., Reiser A., Eachus R. (1988). On the crosslinking mechanism of benzophenone-containing polyimides. Macromolecules.

[26] Hasegawa M., Horie K. (2001). Photophysics, photochemistry, and optical properties of polyimides. Prog. Polym. Sci..

[27] Liaw D.J., Liaw B.Y., Li L.J., Sillion B., Mercier R., Thiria R., Sekiguchi H. (1998). Synthesis and characterization of new soluble polyimides from 3,3’,4,4’-benzhydrol tetracarboxylic dianhydride and various diamines. Chem. Mater..

[28] Rivaton A., Gardette J.L. (1998). Photo-oxidation of aromatic polymers. Angew. Makromol. Chem..

